# Difficult Cases

**Published:** 1888-03-24

**Authors:** 


### DIFFICULT CASES.

[Replies endorsed "Difficult Cases," should be addressed to the Editor.]

Mrs. T. F. Coates, writing from Ewell, Surrey, says:—

"I am desirous of finding a home for a young woman, aged 35, of irreproachable character, whose case is a most distressing and painful one. She has been for many years a domestic servant (cook in a private family), but has gradually lost the use of both arms. As her parents are both dead, she has no means of subsistence whatever. Her sisters, who are themselves in service, would contribute a few shillings weekly, but as this is insufficient for her maintenance I should be glad to hear of an institution that would take in this sad case, and also to receive any practical suggestions or help for her."

"Earlswood": Application should be made to the secretary at the asylum.—[Ed. *T. H.*]

